# A conscious mouse model of gastric ileus using clinically relevant endpoints

**DOI:** 10.1186/1471-230X-5-18

**Published:** 2005-06-06

**Authors:** Matthew A Firpo, Michael D Rollins, Aniko Szabo, Justin D Gull, Jeffrey D Jackson, Yuanlin Shao, Robert E Glasgow, Sean J Mulvihill

**Affiliations:** 1 Department of Surgery, University of Utah School of Medicine, 30 N 1900 E, Salt Lake City, UT, 84132 USA; 2 Biostatistics Resource, Huntsman Cancer Institute, University of Utah, Salt Lake City, UT, USA

## Abstract

**Background:**

Gastric ileus is an unsolved clinical problem and current treatment is limited to supportive measures. Models of ileus using anesthetized animals, muscle strips or isolated smooth muscle cells do not adequately reproduce the clinical situation. Thus, previous studies using these techniques have not led to a clear understanding of the pathophysiology of ileus. The feasibility of using food intake and fecal output as simple, clinically relevant endpoints for monitoring ileus in a conscious mouse model was evaluated by assessing the severity and time course of various insults known to cause ileus.

**Methods:**

Delayed food intake and fecal output associated with ileus was monitored after intraperitoneal injection of endotoxin, laparotomy with bowel manipulation, thermal injury or cerulein induced acute pancreatitis. The correlation of decreased food intake after endotoxin injection with gastric ileus was validated by measuring gastric emptying. The effect of endotoxin on general activity level and feeding behavior was also determined. Small bowel transit was measured using a phenol red marker.

**Results:**

Each insult resulted in a transient and comparable decrease in food intake and fecal output consistent with the clinical picture of ileus. The endpoints were highly sensitive to small changes in low doses of endotoxin, the extent of bowel manipulation, and cerulein dose. The delay in food intake directly correlated with delayed gastric emptying. Changes in general activity and feeding behavior were insufficient to explain decreased food intake. Intestinal transit remained unchanged at the times measured.

**Conclusion:**

Food intake and fecal output are sensitive markers of gastric dysfunction in four experimental models of ileus. In the mouse, delayed gastric emptying appears to be the major cause of the anorexic effect associated with ileus. Gastric dysfunction is more important than small bowel dysfunction in this model. Recovery of stomach function appears to be simultaneous to colonic recovery.

## Background

Ileus is a common post-surgical occurrence characterized by transient impairment of gastrointestinal function. In addition to abdominal surgery, sepsis, trauma, pancreatitis, anesthetic agents, and opioid analgesics are also associated with ileus. The mechanisms of ileus involve neural inhibitory signals and humoral factors including paracrine agents and gut hormones [1-3]. No single event or factor has been clearly implicated as being responsible; it is more likely that multiple mediators act at various times throughout the course of the condition. These mediators communicate throughout the gastrointestinal tract. For example, surgical manipulation of the distal bowel can induce gastric dysfunction [4-8]. Once established, ileus is thought to resolve at different rates in humans with functional inhibition lasting a few hours within the small intestine, 1–2 days within the stomach and 2–3 days within the colon [9,10].

Many recent studies of ileus have focused attention on small bowel smooth muscle dysfunction in isolated muscle strips [11-14]. The weakness of the muscle strip method, however, is the inability to examine complex interactions including brain-gut interactions or organ specific neural reflexes. Similarly, *in vitro *preparations require tissue harvest to assay the condition and do not allow a systems approach to the problem of ileus. The availability of complete genomic sequence information in the mouse has made the application of a systems approach feasible using global gene expression analysis. However, phenotypic characterizations of ileus in animal models that provide system wide information are lacking. Development of such a model in the mouse would be useful.

In the clinical setting, resolution of ileus is determined by the resumption of normal eating behavior and the passage of flatus or stool. Despite the fact that food intake and fecal output are simple measurements of gastrointestinal function they have not been rigorously analyzed in animal models of ileus. Here we describe a conscious mouse model that uses food intake and fecal output to monitor the time course of ileus, allowing measurement of both the magnitude and duration of the condition in the same animal. This integrative approach to defining the phenotype of ileus may facilitate investigations of potential clinically relevant interventions or preventive strategies. In addition, this conscious mouse model offers the opportunity to study molecular events associated with ileus.

## Methods

### Animals

Male C3H mice, 8–12 weeks old, were used throughout the study. Mice were maintained on a 12-hour light/dark cycle and given free access to standard rodent chow and water. For the continuous food intake monitoring study, mice were given 20 mg dustless food pellets (Bio-Serv, Frenchtown, NJ). All procedures were approved and monitored by the University of Utah Institutional Animal Care and Use Committee.

### Food intake and fecal output measurement

Mice were separated into individual cages with free access to water and a tared amount of food. Food, fecal and mouse mass were measured every 12 hours at the beginning of each scotophase and photophase (7 AM and 7 PM). The amount of food consumed over the 12-hour period was calculated as the difference between the mass of food at the end of the period and the amount of food at the beginning of the period. Fecal output was determined from the mass of fecal pellets collected and measured at the end of the 12-hour period. The potential effect of fecal pellet dehydration over the 12 hour period was addressed in pilot studies in which ileus was induced using either 0.1 mg/kg LPS or laparotomy followed by 4 minute bowel manipulation as well as the corresponding controls. Fecal pellets were collected every 12 hours and fecal output was determined in three ways: 1) mass determination immediately upon collection (as reported in this study), 2) mass determination after a further 24 hour dehydration period, and 3) by counting fecal pellets. The decrease in fecal output relative to controls after ileus induction was similar and not significantly different among the three methods of measurement (data not shown). We concluded that the potential effect of dehydration was inconsequential. After a three-day acclimation period, the mice were randomized into experimental groups and a sham group. Data collection started 24 hours before the induction of ileus and continued after induction until a normal (baseline) circadian pattern of feeding and fecal output returned. For continuous food intake monitoring, mice were placed into individual metabolic chambers with free access to water. Single 20 mg dustless food pellets were delivered on demand using a Coulbourn Instruments Habitest system (Allentown, PA) equipped with a pellet feeder and food trough. The presence of a food pellet within the trough blocked light emitted from a light emitting diode reaching a photodetector. When the mouse removed the pellet, a new pellet was delivered. This exchange was automatically monitored and recorded by the Graphic State Notation 2 software program (Coulbourn Instruments, Allentown, PA). Mice were acclimated to the cage for 4 days prior to data collection.

### Induction of ileus

After acclimation and baseline data collection periods, ileus was induced 30 minutes before scotophase unless otherwise noted. To mimic sepsis, lipopolysaccharide (*E. coli *O111:B4, List Biological Laboratories, Campbell, CA) was administered by intraperitoneal injection. The dose-dependent relationship of endotoxin on food intake and fecal output was investigated by determining the time courses for each endpoint after administration of 0.005, 0.01, 0.02, 0.04, 0.1, 0.2, and 0.4 mg/kg mouse mass of endotoxin. Control mice received a similar volume of saline carrier. Post-surgical ileus was induced in mice anesthetized with isoflurane either by laparotomy followed by manipulation of the cecum for 1 minute or laparotomy, evisceration onto a saline moistened sponge and manipulation of the small bowel, cecum and colon for a total manipulation time of 4 minutes. Manipulation was carried out using cotton tipped swabs. After closure of the incision with sutures, the mouse was removed from anesthesia and allowed to recover under a warming lamp for 30 minutes before data collection resumed. To assess the effects of thermal injury, a 20% total body surface area (TBSA) scald burn was induced on mice from which truncal hair was removed. The scald burn was induced by immersion of the exposed dorsal skin in 70°C water for 7 seconds. Immediately following burn injury, the mice were administered 1 ml of intraperitoneal (IP) lactated Ringer's solution. Mice were given 0.5 ml of LR IP every 12 hours for 72 hours post-burn injury. Control animals had truncal hair removed and received a similar time course of anesthesia and resuscitation with LR. Acute pancreatitis was induced in mice after an eighteen hour fasting period using 3 or 7 hourly IP injections of cerulein (50 μg/kg/dose). Sham treated control mice were given similar volumes of carrier (0.1% BSA in PBS). The timing of the injections was coordinated such that the last injection occurred at the beginning of scotophase.

### Gastric emptying and intestinal transit

After a 2-hour fast to empty stomach content, mice were given 200 μl of a 1.5% (w/v) methylcellulose, 0.5% (w/v) phenol red solution in normal saline by gavage. Thirty minutes after gavage, the mice were sacrificed by cervical dislocation, a laparotomy performed and the stomach isolated by clamping the duodenum near the pylorus and the esophagus at the cardia. The entire procedure from sacrifice to clamping was performed in less than one minute. The GIT was removed, separating the stomach from the intestine. The small bowel was dissected from the cecum/colon and divided into four equal length segments by sequential bisection. The amount of phenol red in the stomach and intestinal segments was determined spectrophotometrically after homogenization as described [15]. Gastric emptying was evaluated as the percentage of dye remaining in the stomach relative to the total amount of dye recovered in a standardization group of mice that were sacrificed immediately after gavage. Intestinal transit was determined by measuring the partitioning of dye within the small bowel segments and colon numbered 1–5, proximal to distal. The geometric center of dye transit was calculated for each animal as (∑(% dye per segment X segment number)/100) as described [16].

### Mean arterial blood pressure

Mice were anesthetized with isoflurane and placed on a 37°C heating pad. A polyethylene cannula (PE 10), connected to a pressure transducer, was inserted approximately 5 mm into the femoral artery. Mean arterial pressure and respiratory rate (counted over 1 minute) was recorded every five minutes. After 10 minutes of monitoring to ensure stability of pressure and respiratory rate, LPS (25 μg/ml, in doses of 0.1 and 0.4 mg/kg) or carrier was injected IP. Blood pressure and respiratory rate was recorded every five minutes for a total of 1 hour.

### Activity monitoring

Digital video images were recorded using a personal computer based system consisting of a WebCam (PC-Cam 300, Creative Labs, Milpitas, CA) and WebCam Control Center version 5.6 software . The camera was placed above four standard mouse cages with wire tops. In lieu of litter, a single shredded paper towel was placed in each cage for bedding. A darkroom light equipped with a single 15-watt bulb and a Kodak GBX-2 Safelight filter (Eastman Kodak, Rochester, NY) provided illumination. Individual mice were placed in each cage with free access to water and a tared amount of food. In order to maintain an unobstructed view, water was provided in a glass bottle and food was limited to 4 standard rodent chow pellets (approximately 5 g each). Mice were acclimated to the cages for 5 days prior to data collection. Mice were administered saline, 0.1 mg/kg LPS or 2 mg/kg LPS in a blinded manner 30 minutes before scotophase. Images were recorded at 1 image per second for 10 seconds every 10 minutes for 36 hours. Investigators blinded to the treatment scored the recorded images for mouse activity and mouse induced movement of the food pellets. Activity was scored if gross movement of the mouse body was evident within any of the 10-second images. Likewise, food pellet movement was scored if the position of any of the food pellets were different when the 10 images were compared. New food pellets were placed and food mass recorded at 12-hour intervals corresponding to scotophase and photophase.

### Statistical analyses

Statistical comparisons were made using factorial ANOVA or repeated measures ANOVA (for time course analyses) and Fisher's protected least significant difference (PLSD) post-hoc tests. Comparisons were considered statistically significant at the *P *< 0.05 level. Values are expressed as mean ± SEM unless data from individual mice are shown.

In order to determine the median-effect dose of LPS on food intake and fecal output, the 12-hour (first night) data was fitted to a dose-response curve. The model had an inverse proportionality form with offsets:

*Food intake *= *A *+ (B-A)*D_*m*_/ *(Dose *+ *D*_*m*_*) *+ *error*

where *Dose *is the dose of the drug, *A *is the minimum food intake at arbitrary large doses (horizontal asymptote), *B *is the baseline food intake (when *Dose *= 0) and *D*_*m *_is the median-effect dose, that is the dose at which the food intake is halfway between the baseline *B *and minimum *A*. The *error *term reflects the between-animal variability of food intake, it is assumed to have normal distribution with mean 0 and variance proportional to the response: *error~ N(0,s*^2 ^*(Food Intake))*. The adjustment of the variability depending on the response was necessary as the between-animal variability decreased as the food intake decreased. Since each data point corresponds to a different mouse, the observations are independent. The above model was extended to all time points by modeling the median-effect dose *D*_*m *_as a function of time:

*D*_*m*_*(T) = D*_*m*_*(1) c*^*T*^, where *T *is time in "nights"

that is for each night the dose required to produce a median effect is increased *c*-fold. We also added two random effect terms: between-mouse variability of the median-effect dose *D*_*m*_*(1) *(on log-scale) and of the baseline food intake *B*. These mouse-specific terms allow us to accommodate the within-mouse dependence of the observations.

## Results

### Endotoxin transiently decreased food intake and fecal output in a dose dependent manner

**Figure 1:**
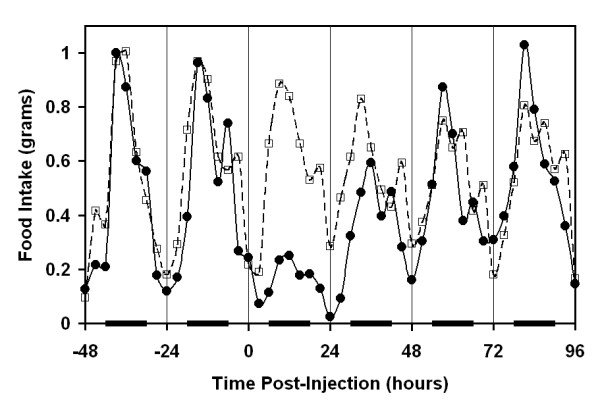
**Circadian pattern of food intake and effect of endotoxin administration. **Food intake was monitored continuously. Endotoxin (0.1 mg/kg, solid line) or carrier (dashed line) was administered IP at time 0. Black bars along the abscissa indicate scotophase. Data points represent mean measurements of six mice in 3 hour time bins.

Untreated (baseline) and carrier treated mice demonstrated a circadian pattern of food intake. Mice took a large meal during early scotophase and a smaller meal near the end of scotophase or the beginning of photophase. Although food intake was lower during the light period, the mice apparently anticipated the pending dark phase as food intake increased near the end of photophase. Administration of endotoxin at time 0 resulted in an immediate and conspicuous decrease in food intake (Figure 1). Feeding behavior began to recover during the ensuing photophase and scotophase with a return to a normal pattern by the third scotophase after injection.

**Figure 2:**
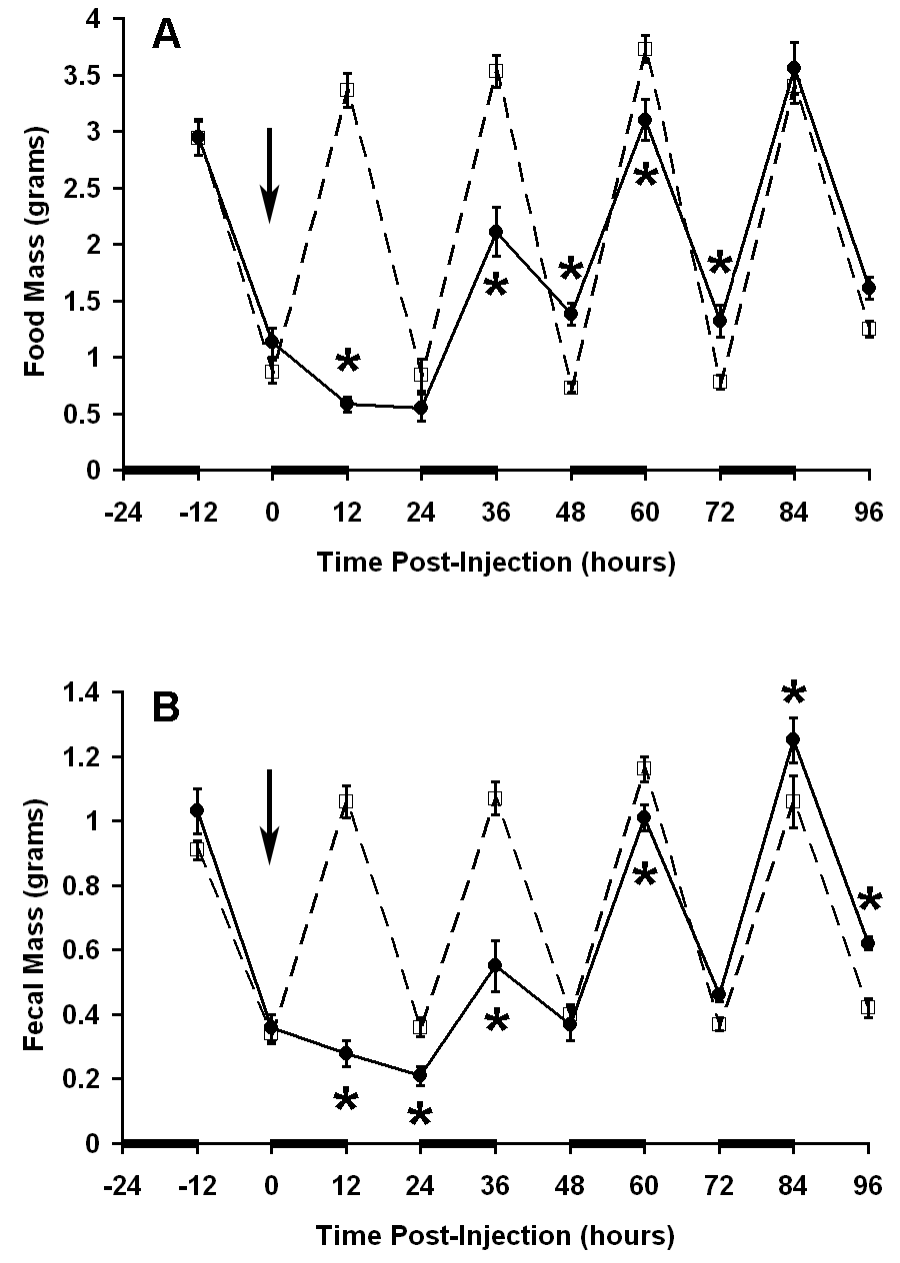
**Induction of ileus after endotoxin administration. **Measurements were made every 12 hours such that each data point represents the food intake (A) or fecal output (B) corresponding to the preceding scotophase or photophase. Bars along the abscissa indicate scotophase. Endotoxin (0.1 mg/kg, solid line) or carrier (dashed line) was administered by intraperitoneal injection at time 0 (arrow). Data points are presented as mean ± SEM (n = 9 mice per group). * *P *= 0.02 vs. control at corresponding time point by repeated measures ANOVA and Fisher's PLSD.

Given the diurnal feeding behavior of the mice with the majority of food consumed during the dark phase, subsequent experiments were carried out by analyzing food intake during each 12-hour photoperiod. This procedure allowed for simultaneous recovery of fecal pellets. LPS administration resulted in a statistically significant reduction in nocturnal food intake during the first (17% of control), second (60% of control), and third (83% of control) scotophases (Figure 2A). Food intake recovered to near normal levels by the third scotophase with complete recovery of the circadian pattern by the fourth scotophase. These data were consistent with the initial findings using continuous food monitoring. Although the amount of food consumed during the first photophase was not significantly different between control mice and LPS treated mice, there was a significant increase in food intake during the second photophase indicating that, during recovery from the insult, the wave pattern of circadian food intake remained dampened. The pattern of fecal output was similar to the pattern of food intake with significantly inhibited fecal output in the first (26% of control), second (51% of control), and third (86% of control) scotophases (Figure 2B). Unlike the food intake data, fecal output was significantly increased relative to controls during the fourth day after injection (116% of control).

**Figure 3:**
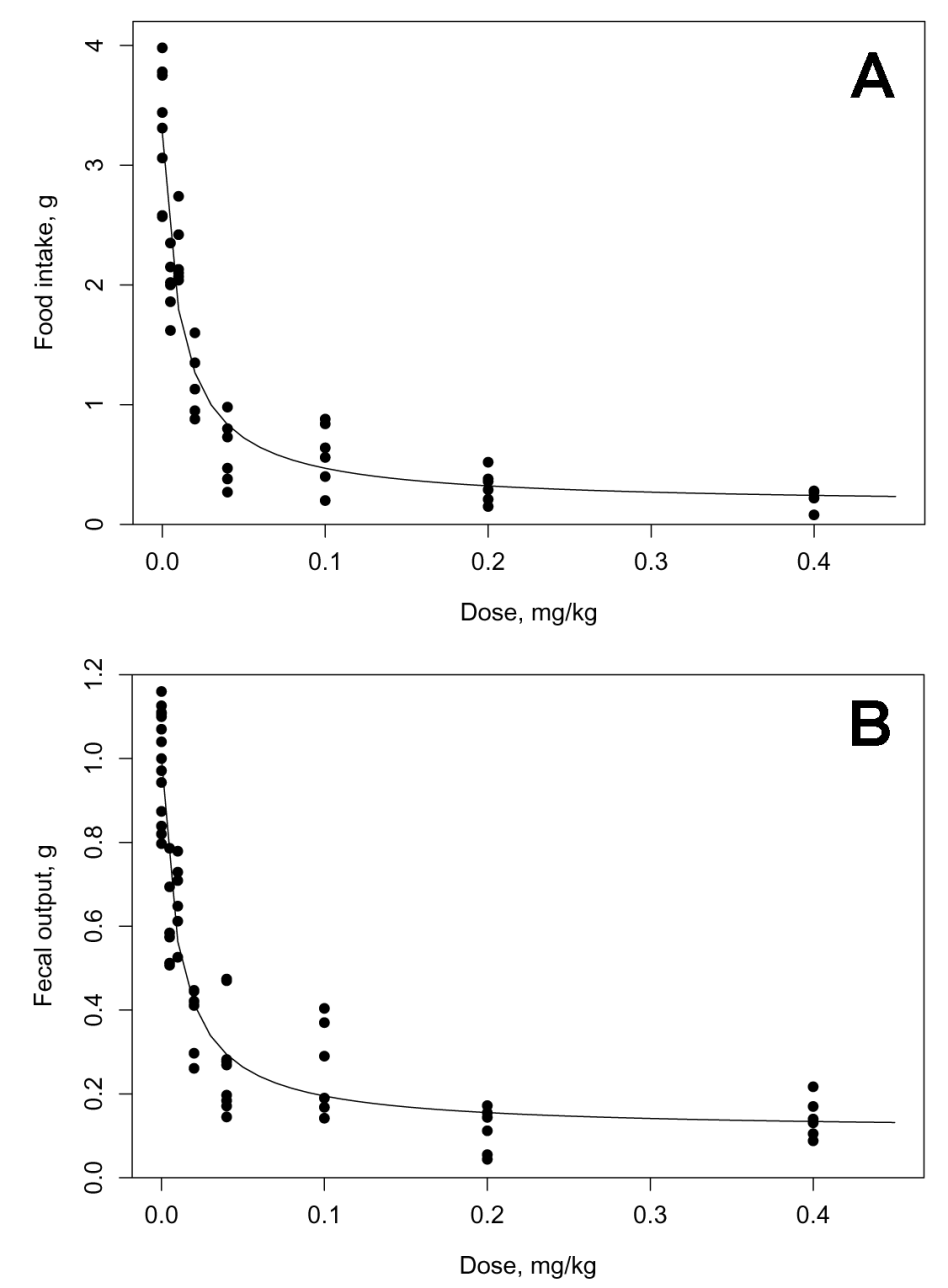
**Effect of endotoxin dose on food intake and fecal output. **Twelve hour food intake and fecal output was monitored over a 4 day period after injection of carrier or various doses of endotoxin. The fitted dose-response curves for food intake (A) and fecal output (B) for the first 12 hours after endotoxin injection are shown (n = 6 mice per dose).

The dose-dependent relationship of endotoxin on food intake and fecal output was investigated by determining the time courses for each endpoint after administration of various doses of endotoxin. Each dose resulted in a statistically significant decrease in food intake and fecal output during the first scotophase, thus, the threshold dose of endotoxin was less than 0.005 mg/kg IP. The estimated parameters and their 95% confidence intervals are given in Table 1 and the plot of the observed data with the fitted dose-response curves are shown in Figure 3. Both fits result in similar conclusions: the median-effect dose *D*_*m *_was about 0.01 mg/kg and there appeared to be a non-zero minimum food intake/fecal output even at high doses.

**Figure 4:**
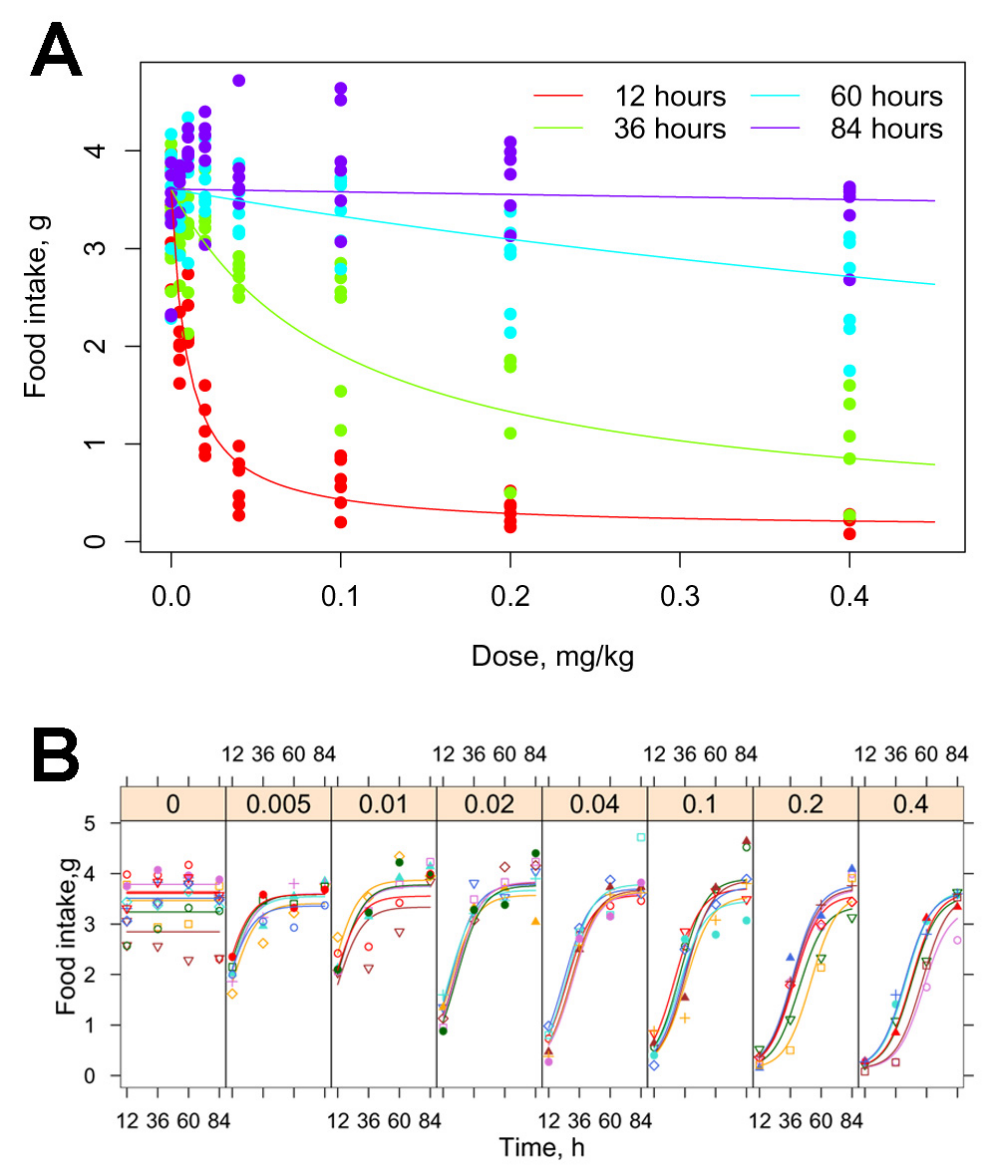
**Effect of endotoxin dose over time.**A. The fitted dose-response curves for food intake are shown for the four nights after injection illustrating the increase in median effective dose each night. B. Time-dependent food intake curves are shown for the individual mice at each dose. (n= 6 mice per dose)

The coefficients of the fitted overall model which examined the data over the first four nights are given in the Table 2. Figure 4A shows the fitted model as dose-response curves for an "average" mouse, whereas Figure 4B plots the time-dependent curves for each mouse separately. The model captures most of the observed phenomena except the "overshoot" on the fourth night (84 hours). A more complicated form would be needed to capture that effect. The resulting c-multiplier indicates that, as the mice recovered, the median-effect dose increased 11-fold each night.

### Decreased food intake and fecal output effects were common to other insults

**Figure 5:**
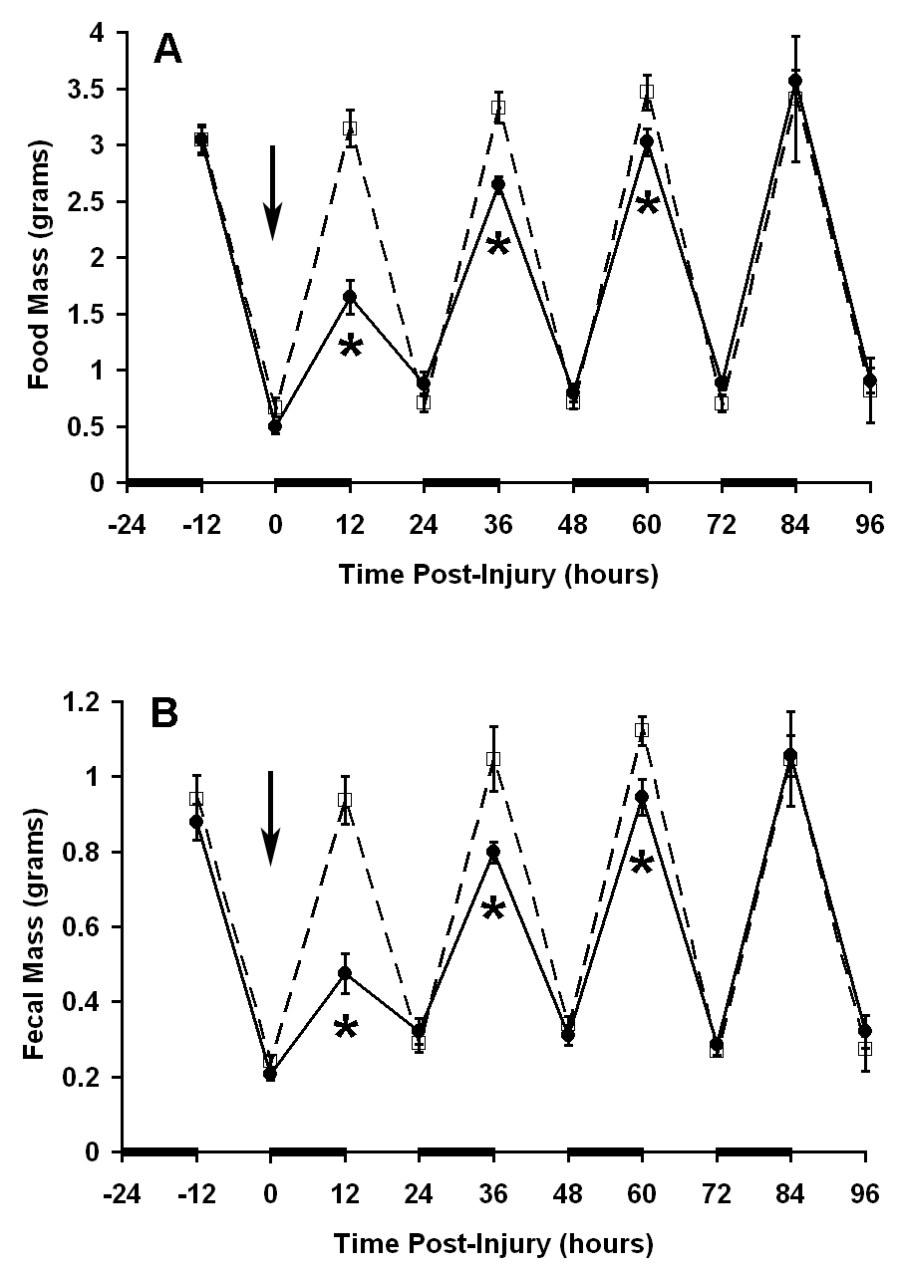
**Induction of post-operative ileus. **Twelve hour measurements are plotted as described in legend for Figure 2. Mice were anesthetized, a laparotomy performed, and the cecum manipulated with cotton tipped swabs for 1 minute at time 0 (solid line). Control mice received anesthesia for a similar time (dashed line). N = 10 mice per group. * *P *= 0.008 vs. control at corresponding time point.

**Figure 6:**
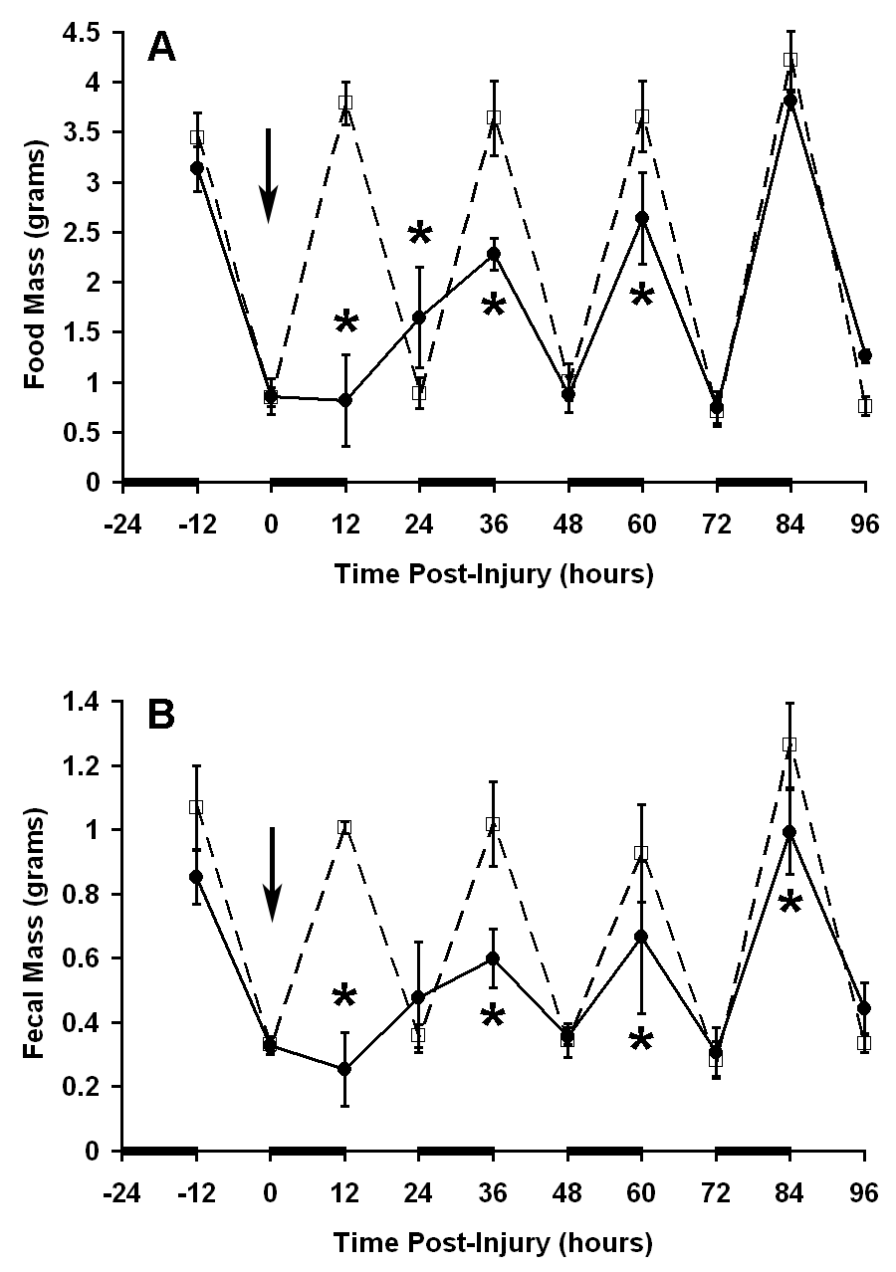
**Induction of ileus after thermal injury. **Twelve hour measurements were are plotted as described in legend for Figure 2. A 20% total body surface area scald burn was induced at time 0 (solid line). Control mice received anesthesia only (dashed line). N = 3 mice per group. * *P *= 0.05 vs. control at corresponding time point.

**Figure 7:**
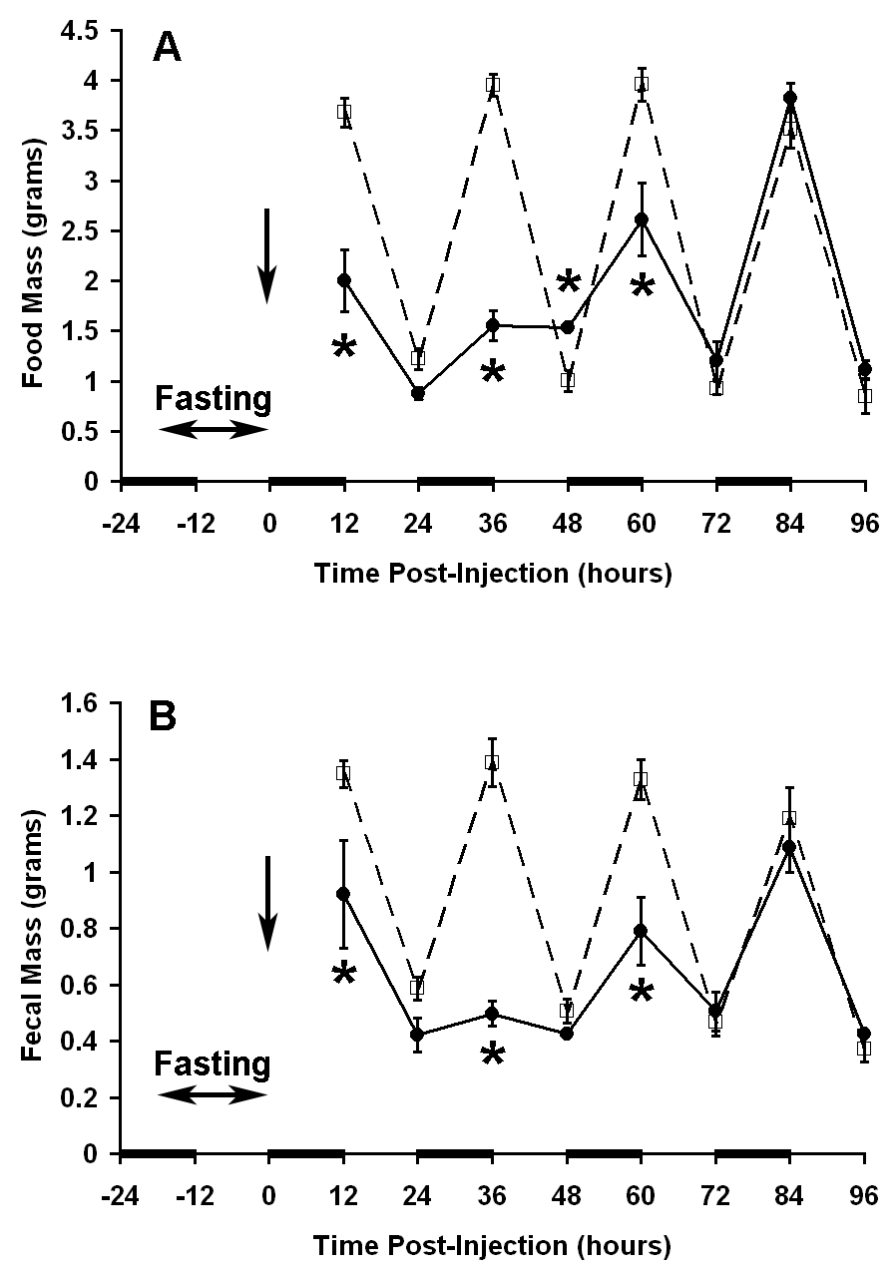
**Effect of cerulein induced acute pancreatitis on food intake and fecal output. **Twelve hour measurements were plotted as described in legend for Figure 2. Seven hourly injections of cerulein (50 μg/kg per dose) were administered after a 12-hour fasting period ending at time 0 (solid line) and compared to control mice receiving carrier alone (dotted line). N = 6 mice per group. * *P *= 0.009 vs. control at corresponding time point.

Other clinically relevant insults that result in ileus were examined using the conscious mouse model. A transient reduction in food intake and fecal output was identified after laparotomy/bowel manipulation (Figure 5), thermal injury (Figure 6), and cerulein induced acute pancreatitis (Figure 7). All three insults caused a significant reduction in food intake (52%, 21% and 54% of control respectively) and fecal output (50%, 25% and 67% of control respectively) within the first 12 hours. The effects of cerulein induced acute pancreatitis induction on nocturnal food intake and fecal output did not reach their nadir until the second scotophase (37% of control for food intake, 36% of control for fecal output) after cerulein injection indicating that, compared to the other insults, more time was required to develop the full effect (Figure 7).

**Figure 8:**
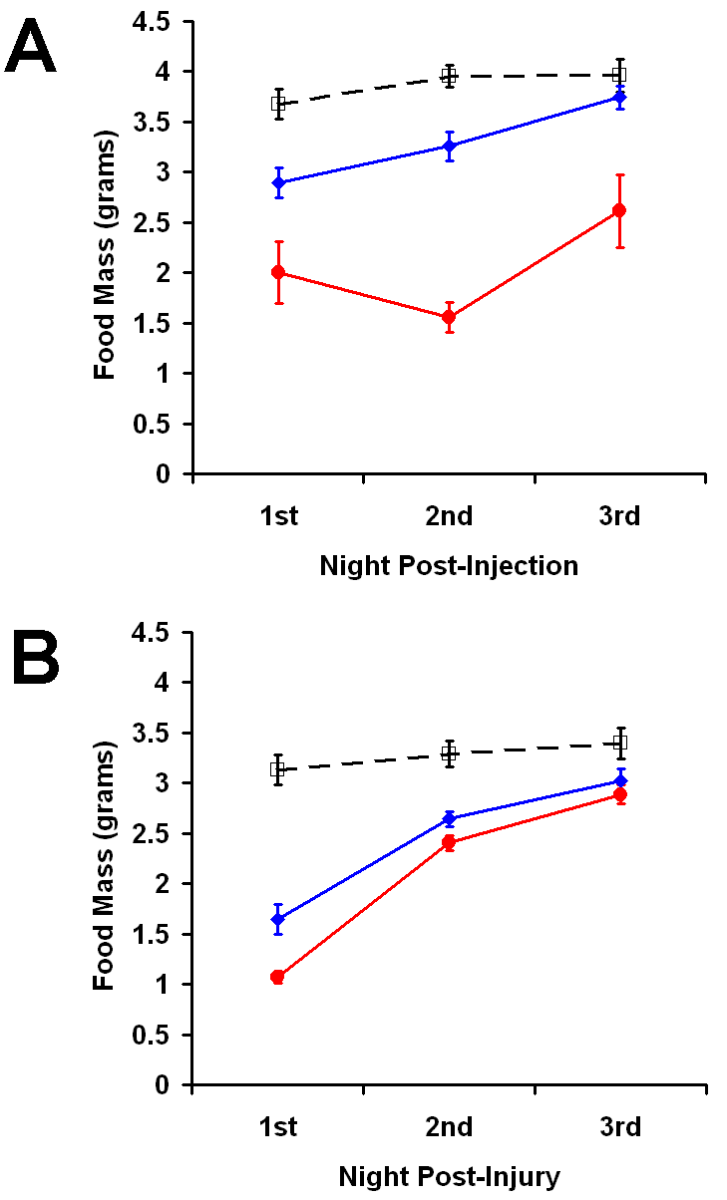
**Correlation of insult severity and the magnitude of ileus.**Nocturnal food intake for the three nights post-insult was plotted for A. after 3 (blue) or 7 (red) hourly injections of cerulein and B. after laparotomy and either a one minute cecum manipulation (blue) or a 4 minute manipulation of the GIT (red). Corresponding control data is shown for each plot (dashed line). Statistical comparisons are discussed in the text. Values are mean ± SEM.

The specificity of cerulein and laparotomy/bowel manipulation for causing the transient decrease in food intake and fecal output was assessed by altering the severity of the insults. As with endotoxin treatment, there was a direct correlation between severity and magnitude of the effects (Figure 8). Acute pancreatitis was induced in mice using a series of either three or seven hourly injections of cerulein. Figure 8A shows the effect of each series on nocturnal food intake over the ensuing three nights. Three cerulein injections resulted in decreased food intake for the first two nocturnal meals (*P *≤ 0.013 vs. corresponding controls) and returned to normal levels by the third night. A more pronounced response was seen after seven cerulein injections, with decreased food intake evident for all three nights (*P *< 0.0001 vs. corresponding control). Statistical comparison of the two insults revealed a significant difference (*P *= 0.0018) at each time point indicating that the magnitude of the response was directly related to the severity of the insult. A similar result was seen when laparotomy/bowel manipulation was used to induce ileus (Figure 8B). Both laparotomy followed by either 1 minute cecum manipulation or 4 minute manipulation of the small bowel, cecum, and colon resulted in a reduction for each of the subsequent three night time meals (*P *= 0.04 vs. corresponding control). Differences between the two insults was not as pronounced as the effect of the cerulein doses, but did result in a significant difference for the first night (*P *= 0.0024).

### Decreased food intake correlated with delayed gastric emptying

**Figure 9:**
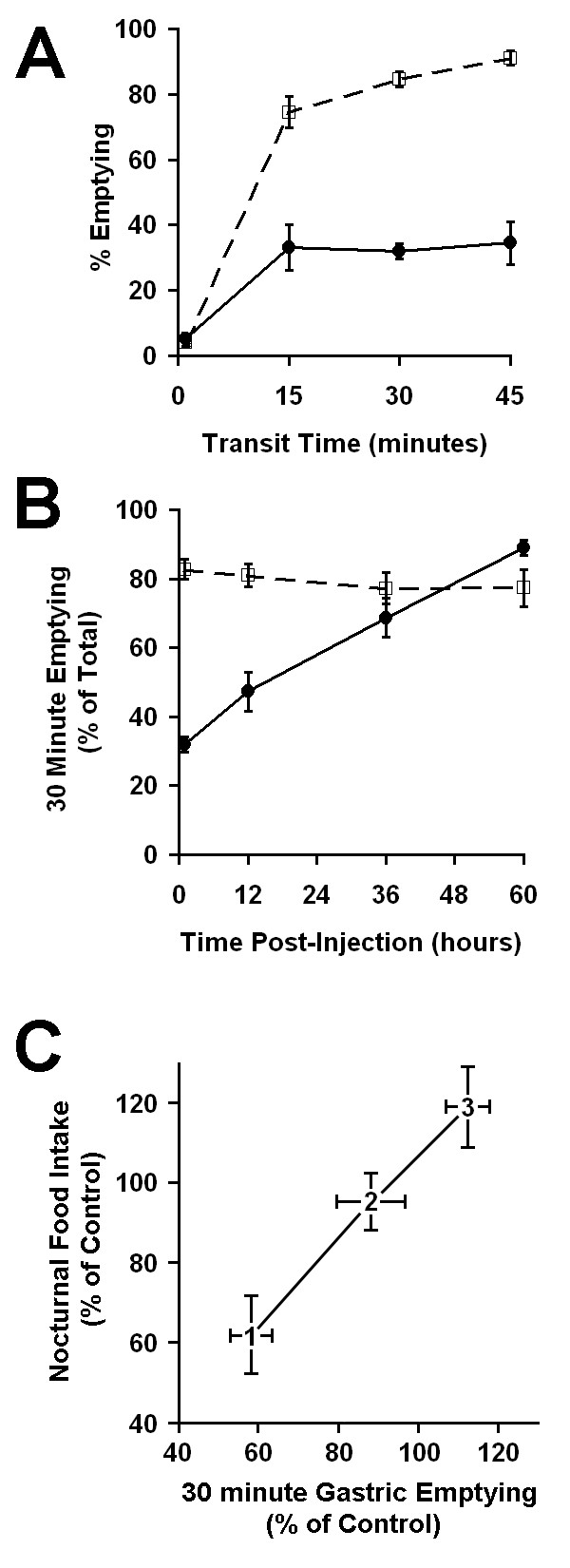
**Effect of endotoxin on gastric emptying. **A. Gastric emptying curve generated 1 hour after endotoxin (0.1 mg/kg, solid line) or carrier (dashed line) injection. A methylcellulose/phenol red dye meal was administered to different groups of mice by intragastric gavage. The amount of dye remaining in the stomach after a 1, 15, 30 or 45 minute transit period was measured. The percent of dye emptied was calculated relative to the total amount of dye gavaged. Each data point represents measurements from 3 – 7 mice. B. The recovery of gastric function was monitored by measuring the 30 minute emptying at various times after administration of endotoxin (0.1 mg/kg, solid line) or carrier (dashed line). N = 3–12 mice per group. C. Correlation plot comparing the recovery of food intake and gastric emptying. Data points represent percentages of the corresponding control. The data point numbers represent the first, second and third scotophase for food intake measurements (ordinate) or the gastric emptying measurement made preceding each scotophase (abscissa). Values are mean ± SEM.

To validate the use of these simple measures as markers of ileus, we examined gastric emptying and intestinal transit in this model. Gastric emptying of a methyl cellulose/phenol red dye meal was measured in mice that had been administered 0.1 mg/kg IP LPS one hour prior to gavage (Figure 9A). A period of rapid emptying was seen in both control mice and mice administered endotoxin over the first 15 minutes after gavage. The amount of dye emptied from stomachs of control mice continued to increase between 15 and 45 minutes after gavage whereas emptying from mice administered endotoxin was static over the same period. Overall gastric emptying one hour after LPS administration was dramatically reduced relative to controls (*P *< 0.0001 vs. corresponding control for 15, 30 and 45 minute time points). The 30-minute rate of gastric emptying was also significantly reduced relative to controls 12 hours after LPS administration (*P *= 0.0053), but recovered to control levels by 36 hours after LPS administration (Figure 9B). The recovery of gastric emptying apparently preceded the recovery of food intake as normal levels of food intake were not evident until the third or fourth night after LPS administration (see Figures 1 and 2A). To graphically evaluate the correlation of the two recovery rates, nocturnal food intake was plotted versus gastric emptying measured 12 hours prior to the corresponding nocturnal meal (Figure 9C). In essence, we asked if nocturnal food intake data could be "predicted" by the antecedent gastric emptying measurement. Both data sets were plotted as percent of control measurements. The resulting slope of one indicates a positive correlation between food intake and gastric emptying.

### Effect of LPS administration on blood pressure

**Figure 10:**
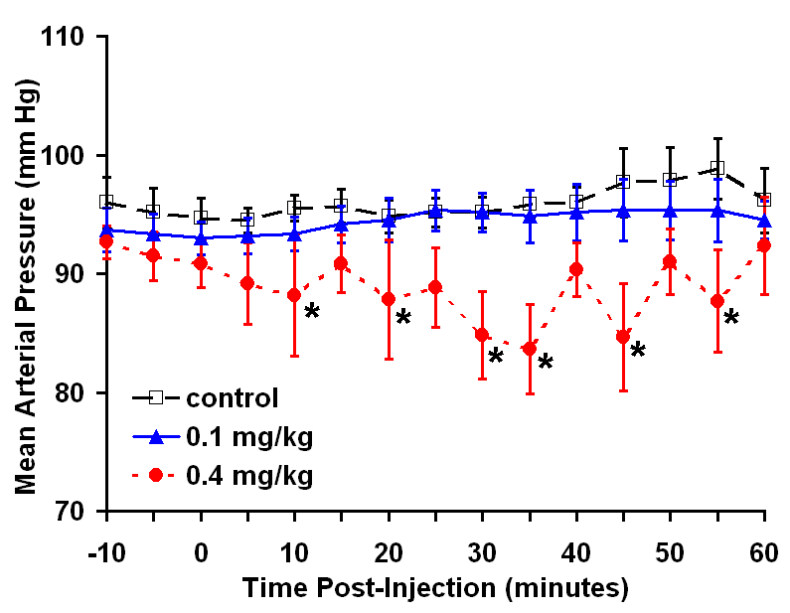
**Effect of endotoxin on mean arterial blood pressure. **Mean arterial pressure was recorded every five minutes. Carrier, 0.1 mg/kg, or 0.4 mg/kg was administered IP at time 0 (n = 6 mice per group). * *P *< 0.05 vs. control at corresponding time.

The decrease in gastric emptying in response to LPS administration was unlikely to be a non-specific hemodynamic effect as mean arterial blood pressure remained unchanged relative to controls for 60 minutes after administration of 0.1 mg/kg LPS (Figure 10) yet a significant reduction in gastric emptying was evident within 1 hour after insult (Figure 9A). Administration of 0.4 mg/kg LPS caused a significant decrease in mean arterial blood pressure at several time points as determined by repeated measures ANOVA. This result suggests that at higher LPS doses, changes in hemodynamics or blood flow may explain, at least in part, functional deficiencies in the gastrointestinal tract. We recognize that measurement of systemic blood pressure may not mirror splanchnic blood pressure or perfusion. In the mouse, however, these latter measurements are technically difficult, highly invasive, and subject to their own set of artifacts related to the procedure.

### Effect of LPS administration on intestinal transit

In contrast to the effect on gastric and colonic function, intestinal transit was not significantly affected by IP injection of 0.1 mg/kg LPS when measured 12, 36 or 60 hours after treatment (Table 3).

### Changes in activity were insufficient to explain decreased food intake

**Figure 11:**
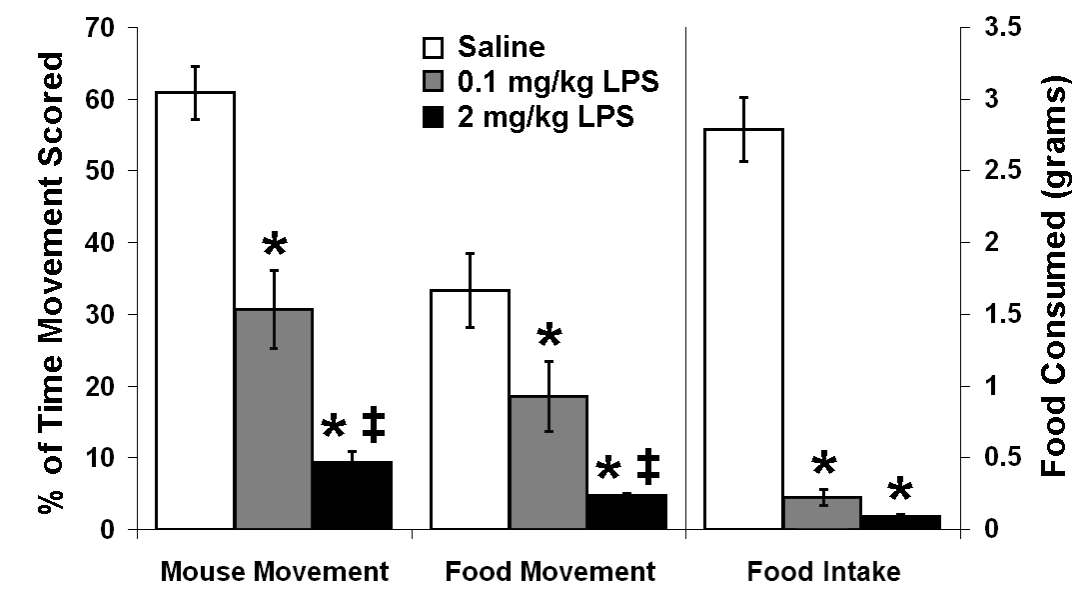
**Activity and feeding behavior during the first scotophase after LPS injection. **Digital video images were scored for activity and mouse induced movement of food pellets during the first scotophase after injection of saline (open bars), 0.1 mg/kg LPS (shaded bars) or 2 mg/kg LPS (black bars) and compared to the total amount of food consumed during the same 12-hour period. Movement data are presented as the percent of the total frames that movement was scored (left axis). Food intake in grams is presented on the right axis. Values are mean ± SEM (n = 5 for saline and 2 mg/kg LPS groups, n = 6 for 0.1 mg/kg group). * P < 0.03 vs. corresponding saline control. ‡ P < 0.05 vs. corresponding 0.1 mg/kg LPS dose.

Mice administered the low doses of LPS described above had relatively normal appearance and activity whereas mice administered higher doses (1–2 mg/kg) showed classic signs of sickness behavior including cachexia, diarrhea, lethargy and decreased grooming. To assess the possibility that the anorexic effect of LPS could be due to changes in activity, including the inability to reach the food pellets, we measured activity during the first scotophase after LPS treatment. Mice administered 0.1 mg/kg or 2 mg/kg LPS showed approximately a 50% and 85% reduction in both general activity and mouse-induced movement of food pellets respectively (Figure 11). Within each dose, activity scores and food pellet movement scores were not significantly different from each other. These data suggest a relationship between feeding behavior and activity level. However, relative to controls, the actual amount of food taken during the first scotophase was reduced to a greater extent than activity (92% food intake decrement for mice administered 0.1 mg/kg LPS and greater than 97% for mice administered 2 mg/kg LPS) indicating that the quantity of food taken during the nocturnal meal was not due to reduced activity.

## Discussion

We have evaluated the feasibility of using food intake and fecal output as markers for ileus in a conscious mouse model by comparing the effect of various insults resulting in ileus on these endpoints. Low doses of LPS, laparotomy/bowel manipulation, 20% TBSA scald burn, and acute pancreatitis comparably produced a transient anorexic effect and delayed defecation consistent with a clinical picture of ileus. The data indicate that both magnitude and duration of the effects were dose dependent establishing a causal link between the initiating insults and the changes in food intake and fecal output. Each insult was self-limiting and mice appeared to have normal grooming habits, vocalizations, and interactions with cage mates within minutes of treatment or recovery from anesthetic. LPS administration in mice has been shown to increase watery secretions in the bowel and induce diarrhea [17,18], either of which would potentially confound our fecal output measurements. However, in our study, we saw no evidence of increased watery secretion and diarrhea was not evident at any LPS dose in which fecal output was measured. A recent study showed that a minimum dose of 10 mg/kg was required to produce a significant increase in watery secretion, as measured by intestinal content mass [17], whereas the highest dose of LPS used in our study was more than an order of magnitude lower (0.4 mg/kg). It might be argued that the inhibition of food intake was caused by the pain and discomfort associated with the various insults. For endotoxin treatment, the delay in food intake directly correlated with delayed gastric emptying suggesting gastric dysfunction was responsible, at least in part, for decreased food intake. Peripheral administration of low doses of LPS is a well established model for sepsis and is associated with the inhibition of food intake in rodents [19]. In rats, the anorexic effect of LPS appears to be mediated centrally through the activity of serotonin [20,21], melanocortins [22], cytokines [23-26], and prostaglandins [25,27]. In mice, the anorexigenic effect of peripheral LPS is attenuated by vagotomy [19] suggesting a brain-gut interaction. There is also evidence that LPS-induced inhibition of food intake in mice is correlated with the inhibition of the orexigenic and motility promoting peptide ghrelin [28] which is synthesized mainly in gastric tissue and acts centrally. Previous studies have shown that low doses of LPS inhibited gastric emptying [29-32]. Here we show that the time course of the LPS induced anorexic effect directly correlated with delayed gastric emptying (Figure 9C). The parallel recoveries of gastric emptying and food intake suggest that the anorexic effect of LPS was in large part due to gastric dysfunction. Furthermore, although administration of LPS resulted in a significant reduction in movement during the first scotophase, reduced activity alone was not sufficient to explain the anorexic effect (Figure 11) as the decrease in the amount of food taken was significantly higher than the decrease in activity. Delayed gastric emptying has also been demonstrated in rodent models of post-operative ileus [1,4], thermal injury [33] and cerulein induced pancreatitis [34].

The common effects suggest, at some level, a common mechanism is shared by the multiple insults. One likely candidate is the inflammatory response since endotoxemia [13,14,35], intestinal manipulation [12,36-38], and ischemia/reperfusion injury [39] have all been shown to promote cellular inflammation within the small bowel and colon in rats. Recently it has been shown in mice that post-operative ileus was associated with inflammatory cell infiltration within the manipulated small intestine, but not the untouched stomach or colon [1]. In the same study, gastric emptying, measured by scintigraphic imaging, was delayed for 24 hours after insult, recovering within 48 hours. This delay was prevented by inhibition of leukocyte recruitment indicating that the inflammatory cells were responsible for delayed gastric emptying, apparently via inhibitory neural signals since hexamethonium and guanethidine normalized gastric emptying. Inflammatory cell infiltration within the pancreas during cerulein induced acute pancreatitis has been well established, however, it is unclear if similar infiltration within the small bowel muscularis also occurs. Such an event would be confirmatory for the model mentioned above. Our data suggests that inflammatory cell infiltration would be delayed relative to other insults since the effect of higher doses of cerulein did not reach its maximum until the second scotophase. Establishment of inhibitory neural signals is also likely to be sequelae common to the various insults. The role of corticotropin releasing factor (CRF) in both appetite regulation and gastrocolonic motor function has been well established [40]. CRF receptors are widely expressed in the brain, notably within brain centers that control appetite, the gastrointestinal tract, and on vagal afferent neurons. The known effects of central and peripheral CRF receptors and ligands makes them intriguing investigative targets for systemic control of the different organ systems involved with ileus.

Ileus is most commonly associated with a transient decrease in gastrointestinal motility. In humans, motility resolves differentially within the gastrointestinal tract with the small bowel recovering most rapidly, followed by the stomach and then colon [9,10] although it is possible that complete recovery of the entire GI tract is not required for clinical resolution. Food intake and fecal output are the most common clinical markers for resolution of ileus and are more generalized indicators of GI function than measurement of motility, MMC or contractility. As shown in this study, delayed food intake is likely to be modified by behavioral effects and thus provides a more integrated description of gastrointestinal function. In our mouse model, fecal output directly tracked food intake for all insults and doses examined in this study. The fitted models for the 12-hour dose-response curves yielded identical median effective LPS doses for food intake and fecal output providing mathematical verification of this correlation. Since data was collected every 12 hours, it is possible that decreased fecal output lagged food intake by a few hours. Therefore it is difficult to distinguish if this tracking was a function of the initiating insult on colonic activity or normal loss of colonic function due to the fasting caused by the anorexic effects of the insults. The fact that the two endpoints tracked demonstrates the functional coordination of the two organs during ileus and illustrates the necessity for endpoints that examine system wide function in order to develop a complete understanding of the mechanisms of ileus. In the small intestine, 30 minute transit of dye remained unchanged between mice administered endotoxin and control mice 12, 36 and 60 hours after injection. This result indicated that if small bowel transit was compromised by LPS, the effect resolved within 12 hours.

The use of food intake and fecal output as endpoints for ileus in mice revealed dissimilarities from more traditional measures of ileus. In the conscious mouse model described here, food intake and fecal output directly correlated with the extent of bowel manipulation. This result is consistent with at least one other study which showed that, in rats, small bowel smooth muscle impairment and inflammation was directly proportional to the extent of bowel manipulation [37]. However, several groups have concluded that inhibition of bowel motility was independent of the degree of manipulation or the duration of surgery [9,41-43]. It is probable that the discrepancy in outcomes is due to the different endpoints measured. A large body of research into the mechanisms of ileus focuses on changes in intestinal smooth muscle contractility. In rats, changes in small bowel smooth muscle contractility after endotoxin administration were seen only above the threshold dose of 5 mg/kg [13]. In our study, food intake and fecal output were responsive to small changes in LPS dose with the lowest dose of LPS tested (0.005 mg/kg) resulting in a significant reduction in both endpoints indicating that they are highly sensitive measures of gastrointestinal function. These data, along with the lack of change in intestinal transit, suggest that low doses of endotoxin produce limited intestinal ileus.

The ability to conveniently monitor the full time course of ileus in the same animal was lacking in most previous models of ileus (although see [44]). The use of food intake/fecal output as simple, clinically relevant endpoints in un-anesthetized mice should facilitate examination of potential treatment strategies. Improvements in either magnitude or duration of ileus are desirable and treatment strategies would be considered effective if they improved either or both. The fitted model described in this study provides a method for quantitative assessment of both magnitude and duration. Changes in magnitude due to a treatment are most easily assessed by measuring the 12 hour food intake/fecal output in LPS challenged mice. Improvement in treated mice relative to untreated LPS challenged mice would quantitatively assess effectiveness. The specific challenge is not limited to LPS. The 12 hour food intake/fecal output measurements for other insults can be expressed in terms of LPS dose by substituting the amount into the formula for the fitted curves. Thus, in our study, a 20% TBSA burn was comparable to a 0.04 mg/kg LPS dose. Although a more involved assay, duration can be quantitatively monitored by the c-multiplier which will be altered as duration changes. In this scenario, an intervention is used to treat mice challenged with several doses of LPS. An increased c-multiplier, then, would indicate a decrease in the duration of ileus and, thus, indicate an effective intervention. Finally, the ability to monitor ileus without the necessity of harvesting tissue will facilitate gene expression studies which require the same tissue for RNA isolation.

## Conclusion

We have demonstrated in conscious mice that a transient decrease in food intake and fecal output are common to multiple insults that result in ileus. Examination of ileus using these endpoints revealed that restoration of gastric emptying preceded recovery of food intake and that gastric and colonic function recovered nearly simultaneously. The easily monitored, clinically relevant endpoints provide a convenient means for examining ileus in a mouse model and should prove useful for analysis of potential intervention strategies and mechanistic investigations of ileus.

## Competing interests

The author(s) declare that they have no competing interests.

## Authors' contributions

MF coordinated the study, assisted with data collection, carried out data analysis, participated in the study design and drafted the manuscript. MR carried out the pancreatitis experiments and assisted with the transit experiments. AS participated in study design and performed statistical modeling. JG carried out the activity monitoring experiments and assisted with food intake and fecal output data collection. JJ assisted with the activity monitoring experiments and food intake and fecal output data collection. YS performed the laparotomy/bowel manipulations and burn injury animal models, blood pressure monitoring, tissue harvest, and assisted with food intake and fecal output data collection. RG participated in study design, coordination, and data review. SM conceived of the study, participated in the study design, supervised data analysis and edited the manuscript. All authors read and approved the final manuscript.

## Pre-publication history

The pre-publication history for this paper can be accessed here:



## Figures and Tables

**Table 1:** Parameters of the 12-hour endotoxin dose-response curve. Results of the fitted model examining the effect of various doses of endotoxin on food intake and fecal output during the first scotophase are shown.

**Parameter**	**Estimate**	**95% Confidence Interval**
*Food Intake*		
*A *– minimum, grams	0.16	(0.08, 0.24)
*B *– baseline, grams	3.27	(2.90, 3.64)
*D*_*m *_– median effect dose, mg/kg	0.011	(0.007, 0.015)
*s *– standard deviation, grams	0.33	(0.28, 0.41)
*Fecal Output*		
*A *– minimum, grams	0.11	(0.08, 0.14)
*B *– baseline, grams	1.00	(0.88, 1.12)
*D*_*m *_– median effect dose, mg/kg	0.010	(0.006, 0.015)
*s *– standard deviation, grams	0.13	(0.11, 0.16)

**Table 2:** Parameters of the endotoxin dose-response curves for food intake over time. Results of the fitted model examining the effect of various doses of endotoxin on nocturnal food intake extended to include the four nights after LPS injection.

**Parameter**	**Estimate**	**95% Confidence Interval**
*A *– minimum, grams	0.13	(0.05, 0.21)
*B *– baseline, grams	3.50	(3.61, 3.72)
*D*_*m*_*(1) *– median effect dose for the first night, mg/kg	0.010	(0.007, 0.012)
*c *– multiplier of the median effect dose for each additional night	11.00	(8.62, 14.04)
*s*_*B *_– standard deviation of *B, g*	0.27	(0.18, 0.39)
*s*_*D *_– standard deviation of log*D*_*m*_*(1)*, g	0.47	(0.33, 0.66)
*s *– residual standard deviation, grams	0.23	(0.20, 0.26)

**Table 3:** Intestinal transit of methyl cellulose/phenol red dye after endotoxin injection. The 30-minute transit of dye was measured in each of five intestinal segments and the mean geometric center calculated. (n = 6 for each group, *P *= 0.3 by two-way ANOVA)

**Treatment**	**Time Post-LPS Injection**		
	12 Hours	36 Hours	60 Hours
Control	2.28 ± 0.17	2.58 ± 0.16	2.36 ± 0.19
0.1 mg/kg LPS	2.59 ± 0.10	2.71 ± 0.09	2.50 ± 0.12
